# Tricks of the Psychotherapist's Trade
*A version of an address to the Bristol Medico-Chirurgical Society on 8th April, 1964.


**Published:** 1964-07

**Authors:** D. Russell Davis


### TRICKS OF THE PSYCHOTHERAPIST'S TRADE*

BY

D. RUSSELL DAVIS

A boy is learning to swim. He is swamped by an unexpected wave, and comes frightened out of the sea. The psychotherapeutic task is to get him back into the sea under conditions in which he can succeed where previously he failed.

The popular belief is that the sooner the rider who has taken a toss gets back into the saddle, the more readily he will do so. There is probably some truth in it, although there is no systematic evidence on which to decide. Similarly, the driver, or the pilot, or the workman, who has sustained an accident, regains his confidence more easily, it is generally supposed, if he resumes soon. The student who has fled from the examination room can more readily be got back into it again the same or the next day than if his return is delayed. The spouse who has left home after a quarrel can sometimes be persuaded to return to the home immediately; the longer the delay, the greater the difficulties. The psychotherapist supposes, therefore, as a general rule, that his task will be easier if the boy can be got back into the sea immediately. Otherwise defences become established, and harden.

The boy may be said to suffer from an "approach-avoidance" conflict. Most fathers try first to strengthen the approach tendency by making swimming appear more attractive or by offering bribes. When these measures fail, as usually they do, they try to weaken the avoidance tendency by punishing it, e.g. by showing disapproval or through sarcasm. They then ring the changes from suggestion to persuasion, exhortation, cajolement and coercion. They are encouraged in these efforts for a while because they very nearly prove effective, although not quite. The boy may be got to the edge of the water, but not into it, for, in accordance with the principles of learning theory, the closer he gets to the danger, the stronger is the avoidance tendency. At the edge of the water, the boy's fear is intense. At this point some fathers resort to the method of "forced solution", i.e. the sink or swim method, and push, drag or throw the boy in.

### DIRECT METHODS

The consequences of forced solutions are not always advantageous. The boy does sometimes sink. The psychiatric casualties in war-time who were forced back into the front line sometimes wandered off in a fugue or threw their lives away. The pilot who returns to flying immediately after an accident may be clumsy and inefficient. The student forced back into the examination room becomes disorganized or depressed—one of my patients last year so treated, merely wrote out twenty times on his paper the words: "Nihil sum". The young man who gets married in order to overcome sexual difficulties may become seriously disturbed.

Forced solutions are seldom practicable, for sufficient pressures cannot be brought to bear on the patient. Moreover, he can usually find a way of escape, or find a more sympathetic doctor. Anyone who has attempted a forced solution in a case of anorexia nervosa knows of the lengths to which the patient will go to evade pressures, and the variety of the devices to which he, or rather she, is prepared to resort. Forced solutions are sometimes possible, however, in the special circumstances of active duty in war-time. A student shrinking from his examinations may sometimes find it difficult to escape from his friends. Young men do get hooked. A special instance of forced solution is the administration of a narcotic intravenously in order to revive the memories

*A version of an address to the Bristol Medico-Chirurgical Society on 8th April, 1964.

of a traumatic experience. The patient's defences are removed in this way; through recall he re-enters the danger situation—unless he falls asleep.

Lesser degrees of coercion are used very often, both inside and outside departments of psychiatry. They are the essential component of all methods intended to make the patient face his problems. Yet direct methods of this kind are of limited value, and are often ineffective when defences have become established. Nevertheless we all resort to them with an optimism which is not justified by the results they achieve.

### LEARNING IN EASY STAGES

The psychotherapist's trick is very different. The first thing he does appears at first sight to go against common sense. He gives back to the patient as much control over the situation as possible. He removes all coercions and leaves the patient entirely free to retreat from the danger situation. This method allows the boy to approach the sea without any pressure from behind, so that he feels free to run away as soon as he becomes afraid. In these circumstances he will make tentative explorations without fear. If these are successful, they will increase in extent and duration. No more is then necessary than to ensure that he does not again meet a situation he cannot easily master. His confidence is gradually restored through success in his tentative explorations. Gradually extending their range, he re-enters the sea. All this may happen after his father has given up the direct method in despair.

The psychotherapist's method has several advantages. It places the responsibility on the patient to decide and act on his own behalf. The psychotherapist's role is largely passive. The method depends upon learning in easy stages, gradualness being an essential feature. It aims at building up confidence through success and thus conforms to the best educational practice. Not least in importance, it has been shown in animal and human experiments to be an effective method.

Note that psychotherapy, as I have illustrated it, consists in creating the conditions in which behaviour can be modified through experience. It can take place on the beach or airfield or anywhere else. Its methods are not exclusively or even mainly verbal. Its theory is not esoteric. The relationship the psychotherapist forms with his patient is no different from that which a gentleman in the best English tradition would wish to form with an equal. My main illustration—that of the boy who shrinks from the sea—suffers from one defect. I do not know of any systematic observations on psychotherapy in a substantial series of cases of this kind, however large the store of unsystematic observations there may be to call on. I chose it in order to avoid detailed discussion of the artificial laboratory situations in which experimental observations have been made.

There are many laboratory experiments to justify the points I have made. In my own experiments, for instance, subjects were set to perform on an artificial task demanding a high level of skill. The level of difficulty was raised fairly quickly so that the subjects were caused to lose control over the task. After breaking down in this way, they showed a strong aversion for the task and were unable to perform at a level of difficulty at which previously they had been able to manage quite well. The best way of restoring their capacity was to provide opportunities for them to do so, and to lower the level of difficulty to a point at which they could readily succeed. The level of difficulty was then raised gradually, all the time making sure that they did not fail.

### CLINICAL APPLICATION

The same principles can be applied in clinical work. Consider a case of hysterical amnesia of recent onset. Many would try first to remove the amnesia by direct questioning, but this is rarely if ever effective. Often it increases the range and degree of the amnesia. The psychotherapist proceeds by opening up communication with the patient on trivial matters which do not evoke anxiety, such as immediate circumstances in the ward, or even the weather. If he asks questions at all, they are capable of being answered by the patient easily and without embarrassment. He allows the patient to extend the range of the discussion, and to retreat from any point which might cause anxiety. He reassures by showing that he accepts without reproof what he is told, and in this way builds up the patient's confidence in his good will. When this process has gone far enough, the patient will begin to speak of matters of real importance. All this may take no more than a few minutes; usually several short sessions are required, spread over a few days. However rushed he may be, the psychotherapist must convey the impression that he has plenty of time. This method is better than using drugs to remove defences, for it helps to establish a good relationship with the patient, and does not make loss of memory appear mysterious. A similar method may be used in the treatment of hysterical aphonia and other hysterical losses of function, with results which are better and more lasting than those obtained by "vigorous" methods. The essence of the trick is to start by giving the patient something to do which is very easy indeed.

There is nothing new in the method. All I have done is to express in modern terms ideas which are implicit in Freud's method of psycho-analysis. The psycho-analyst removes all the pressures on the patient to recall, and leaves him free to recall what he will when he will. There is one difference of great importance. In Freud's method the patient is allowed, indeed encouraged, to regress to a state of child-like dependence on the psycho-analyst, but in the method I describe he is encouraged from the beginning to adopt a mature and responsible rôle in his relationship with the psychotherapist. Greater knowledge of the processes involved enables the modern psychotherapist to achieve results without the extravagance in the expenditure of time that was necessary in Freud's method.

"Behaviour therapy" requires the psychotherapist to take a much greater degree of control over the interview and to make demands on the patient to do certain things. This is a crucial difference from the method I have described, and it is one reason why behaviour therapy has not come into general use. Except for this difference, Wolpe's method of desensitization is very like the one I have described. Also, behaviour therapy is derived from a specialized theory of learning, and one which has, I think, been superseded. The evidence cited by Wolpe, Eysenck and others as proof of its efficiency has not been regarded as reliable.

The problem in the case of the boy is that experience induced in him an attitude towards the sea which precluded further experience of the sea. This attitude was fixed therefore, and was only modified when further experience of the sea was contrived. The psycho-analytic problem is similar. A traumatic experience becomes "fixated" in memory because repression prevents its recall. Not being recalled, it is not modified by further related experience. In health, attitudes towards persons, events and situations undergo continuous modification and elaboration through new experience. A characteristic of neurosis is that it prevents new learning.

Another clinical example—a child is refusing to go to school. The difficulties tend to increase if there is delay in getting the child back to school. Attempts at a forced solution nearly always fail. The best chance of a quick result lies in gaining the child's confidence and in encouraging him to decide for himself to return. But the psychotherapist's efforts may be negatived by the mother. Treatment of the patient only is not enough. Account has also to be taken of his family.

This is usually necessary in cases of depression. A patient becomes depressed as a result, let us suppose, of stresses arising out of his relationships with his family. Depression may be regarded as a complex reaction to a traumatic experience. Let us suppose that the crucial experience in this case is a quarrel with his wife. He has retreated from his relationship with her just as the boy retreats from the sea. The psychotherapist's task is to create the conditions in which he can become reconciled with her. Admission to hospital, which represents the removal of the pressures to return to her, may result in clinical improvement, but he only recovers fully, not while still in hospital, but when he has managed to return home. Treatment in a modern psychiatric unit places no restrictions upon visits by his wife, during which they may make tentative explorations of each other. It also permits him to make visits to his home, brief to begin with and to be cut short if he feels threatened. E.C.T., perhaps medication, and discussions with him and his wife relieve anxiety in various ways and help him to try again. If things go well, confidence, quickly destroyed by the traumatic experience, is restored slowly but progressively. The degree and permanence of his recovery depend upon the degree to which he acquires through learning new habits in his relationships with his family. The patient thus has lessons to learn if he is to recover. The psychotherapist's rôle is that of tutor.

### GENERALIZATION AND LOCALIZATION

A new illustration will help to introduce some new problems. Consider a student who has failed an examination, let us suppose, in pharmacology. From this experience he may draw the conclusion that he has not yet learnt enough pharmacology, or that he is not much good at pharmacology, or at examinations or at academic work, or that he is stupid or lazy or in some other way not up to the standards required by the medical school, or that he is a failure as a student or as a person, or that nothing he does is likely to be any good, or that he is of poor stuff or bad or wicked. This is a series of conclusions of increasing generality and increasing depressiveness.

The student may alternatively conclude that the examiners have made a mistake, or that they are penalizing him for some other misdeeds, or that they are against him for some other reason or, at the extreme, that they are all in league with others to persecute him. This is a series of increasingly paranoid ideas. With them tends to go a belief that he is good or very good at pharmacology.

Paranoid ideas are relatively uncommon in medical students. Some degree of depression, on the other hand, is the rule. Nearly every student who has failed an examination entertains doubts about his capacity and his goodness. Similarly the boy who has fled from the sea will entertain doubts about his courage and whether he is loved or is lovable. These doubts are increased if his father uses a direct method and punishes the avoidance tendency. Almost certainly the father will then show disapproval and impute cowardice.

There is of course a strong tendency to generalize from a failure, even if it is a failure in a limited respect only, and the generalization may have a degree of validity, but not always so. Who does not generalize when his car is running badly or the wind-screen wiper is not working, and regard it as a thoroughly bad car, when it is really a reasonably good car with a dirty distributor or a defective wiper?

The most specific conclusion—the one which most clearly localizes the badness—tends to be the most favourable because it indicates the way in which things can best be put right. The student can get out his pharmacology books. The boy can learn to swim. The distributor can be cleaned, or the wiper mended. Depressive ideas are less readily corrected. Moreover, they distract attention from the specific task, and handicap performance in it, and divert energies into the seeking of reassurance. We all recognize the difficulties people make for themselves when they are unsure of themselves or have inferiority feelings. They tend to be dependent upon others, and seek their support, and cannot easily be got to settle down to a job. We know too of the variety of ways in which students deal with their depressive feelings after they have failed an examination. Some, for instance, seek relief in pubs, a few in sexual adventures, and even a few nowadays get a prescription from their doctors for one of the new psycho-tropic drugs.

Similar behaviour was observed in those subjected to failure in the laboratory experiments. Most of them deprecated or reproached themselves. A few blamed conditions of the test, or the apparatus, or even the experimenter. With feelings of both kinds tended to go inattention and failure of concentration. The capacity of those expressing depressive ideas tended to be restored relatively easily, in the manner already described, if the level of difficulty was dropped sufficiently to take account of the poorness of their concentration to start with. It was more difficult to restore the capacity of those expressing paranoid ideas.

Depressive feelings after an examination failure tend to be short-lived. Usually the student is quickly reassured by his friends and teachers, and his optimism is revived when he gets back to work. Paranoid ideas fade less quickly and are less easily dealt with.

There are other types of case in which depressive feelings are more persistent and more troublesome. The psychotherapist then tries to do two things. Firstly, he reassures; i.e., he tries to restore the patient's good feelings about himself. I shall use the term "reassurance" in this sense, and not to mean the attempt to dispel apprehensions about the future. Secondly, the psychotherapist tries to localize the badness, and thus to reduce the tendency to generalize, and also to show what has to be done. Efforts in these two directions reinforce each other.

Reassurance plays a main part in all psychotherapy, whatever other processes are involved as well. We approach now some difficult problems of great importance to the psychotherapist, as well as to ministers of religion and others. There are no generally agreed solutions, and therefore no clear rules for the psychotherapist to follow. What I have to say is a personal opinion. In my view, several things need always to be conveyed to the patient. (1) He is not in any sense an outcast, and is accepted as an ordinary member of the community, i.e., as one deserving of the three As—acceptance, approval and affection. (2) His distress is recognized. (3) No punishment is to be imposed. The psychotherapist is not prevented from condemning or expressing disapproval of a behaviour, but when he does so, his purpose is to convey information about his views of the character of the behaviour, and not to inflict pain or penalty or to cause the patient to suffer. (4) He is to be treated as fully responsible, and is expected to decide and act on his own behalf. This is only another way of saying that as much control over the situation as possible is given back to him. There are some circumstances in which a patient has to be treated as irresponsible in certain respects. He has then to be told what decisions have been taken out of his hands. By defining these circumstances, the Mental Health Act makes it clear that normally the patient is to be regarded as responsible. (5) He is to be helped, if he wishes it, to discover what is wrong, and what has to be put right; or he is to be helped, if he wishes it, to decide in what way restitution should be made.

### MAKING A DIAGNOSIS

In the examples we have discussed so far, the depressive feelings have been known to arise out of such experiences as being swamped by a wave, failing an examination or failing in a laboratory test. However, there are many clinical cases in which their origins are obscure when the patient is first seen. Some psychiatrists then attribute them to endogenous factors, and make no further attempt to define their causes Others think it reasonable to suppose as a working assumption that, because they in many cases lie in recent experience, they do so in all cases. There is nothing in my clinical experience which makes me wish to reject this assumption. There are very few cases in which causes for depression, and especially for the persistence of depression, cannot be found after the patient has been seen several times. The identification of the causes is what I have referred to as localizing the badness. Making a diagnosis would be a more conventional way of putting it.

One of the most helpful things a doctor does is to specify what is wrong. Waking up feeling ill, with aches and pains all over, a patient feels that he is disintegrating and rotten all through. Even if it is of poor quality, a diagnosis—a slight touch of 'flu, for example—relieves anxiety because it places limits upon generalization and makes the patient feel better about himself. Again, a mother who brings to the doctor an enuretic child, for instance, or a child having difficulty in learning to read, may say in effect: "He's a bad child. He stinks". Or "He's lazy". To this the paediatrician replies: "No, Madam, he's a good child, but he has a weak valve in his bladder, or an inborn defect in the superior convolution of the temporal lobe of his dominant hemisphere", or whatever else fits with the theory he is currently subscribing to. Paediatricians are inclined to "somatize" the defect. This is an effective way of relieving anxiety, but has the disadvantage of diminishing the patient's feelings of responsibility for the control of the symptom. Reassurance may be effective even if the aetiological views expressed are fallacious. But the most effective reassurance is based upon a correct appraisal of the problem. On the other hand, the doctor who has a little more knowledge of child psychiatry may say in effect: "No, Madam, he's a good child, but he has a bad mother", which is helpful neither to the child nor his mother. The psychotherapist gives over the child the responsibility of managing and eventually gaining control over his disability.

Depressed patients show a strong tendency to localize the fault spontaneously. A depressed patient may attribute his distress to a particular thing about his house, for instance, or the food he has been eating. While he does so, he may feel rather better. But when he puts right what appears to be wrong, he gets little benefit, or rather the benefit is short-lived. Depressed patients commonly change their houses, their glasses, their jobs, or their spouses. More often, however, they tend to somatize, i.e., to localize their distress in a bodily symptom.

### PERCEPTION IN ANXIETY

A certain amount is known about the ways in which anxious patients draw conclusions about the nature of the chaotic sensations they experience. In brief, their perception of them is unstable, in fact kaleidoscopic. Consider first a timid child in a partially darkened room at night. Behind the door he sees a wolf, or a witch or a troll. When the light is turned on, he sees that the shadow is his dressing gown. This illustrates a general tendency in perception. Even when sensory information is incomplete, an interpretation is placed upon what is seen or felt. In other words, a hypothesis is formulated, which is modified or discarded as soon as more information becomes available. The tendency is stronger when the anxiety level is high. Walking along a lonely road on a dark night, a confident man is able to suspend judgement about the masses of light and shade he sees. The nervous girl perceives them as foul fiends or a gang of "Teds" about to pounce on her. The higher the level of anxiety the more rapid in some circumstances is the shift from one hypothesis to another.

Each hypothesis provides an organization into which for the moment all the data fit. An anxious man who wakes at night and becomes aware of vague pains interprets them. For the moment everything fits into the hypothesis that he has a coronary, and now renal colic, or perhaps appendicitis. In these circumstances he is highly suggestible, with the result that a certain hypothesis appears confirmed and is stabilized. When it is refuted by further information, the kaleidoscope is turned, and all the data now fit into a new pattern, a new hypothesis, a new diagnosis. Each new diagnosis is accepted with some sense of relief, because it restricts generalization, localizes and reduces uncertainty.

### SOMATIC SYMPTOMS

A depressed patient who localizes his distress in bodily symptoms is likely to arrive sooner or later in the medical or surgical out-patients, where he is in grave danger of being investigated and treated for his symptoms. For a while, he does not give utterance to his depressive feelings. Or, if he does, his doctors are so busy peering into whatever orifice they specialize in that he is not heard. But the benefit is short-lived, the kaleidoscope turns, and a new localization is found, which brings about referral to another department of different interests. Hope is renewed, only to be dashed again. Eventually his notes achieve the critical thickness which justifies referral to the psychiatric department. Some patients get there more quickly; the unco-operative, and the ungrateful, and those who mention suicide.

I have laboured the discussion of the perceptual processes in the anxious because when patient and doctor abandon one hypothesis and go over to another, the patient tends to be judged harshly. The largely meaningless word "hysteria" is likely to be applied to him, and various unworthy motives are imputed. Yet the shift of hypothesis, or the failure to shift, is due to processes which go on in everyone. Admittedly, the patient is usually more suggestible than are his doctors.

### BIOLOGICAL AND PSYCHO-SOCIAL EXPERIENCE

The psychotherapist's trick is a straightforward one. Like the physician or surgeon who is investigating a case already investigated and treated unsuccessfully by someone else, he assumes that whatever went wrong at the start of the illness has not yet been put right. Like them he enquires into the events and circumstances which immediately preceded the illness. But he does not restrict his enquiries to the physical and biological. On the contrary, he extends his questions to psycho-social events, and asks not only what biological experiences did the patient have, but also what psycho-social ones.

Being swamped by a wave rarely has anything to do with it; failing an examination, less rarely. Mostly the relevant experiences arise out of crises in relationships in the family, such as quarrels with or bereavements of a person of importance to the patient; a parent or boy-friend or girl-friend, a spouse or a child, more often a daughter than a son. There are usually good reasons for supposing that the patient has been sensitized by previous experience. A crisis brings about changes in the patient's life-situation, or world of persons, which demand from him a new adaptation. Illness reflects the failure to make a satisfactory adaptation.

It is a constant source of surprise to me how little interest physicians and surgeons have taken in their patients' psycho-social experience. Meticulous comprehensive enquiries into physical and biological experience seem often to go with complete neglect of psycho-social experience. Yet the effects of the loss of a spouse, for instance, produces very great effects upon almost all organic functions through a variety of mechanisms.

Identification of the relevant psycho-social experiences leads on to the discussion of the adaptations demanded of the patient. Very often the situation to which the patient has to adapt is intractable. It is generally supposed that talking about the difficulties makes them more tolerable, even when it does not make their solution any easier.

The psychotherapist's objectives are limited. They are to create the conditions in which behaviour tendencies can be modified and new learning can take place. He has plenty of opportunity to hear about the miseries and hardships of life, but he does not try to change the patient's situation. On the contrary, he has to get the patient to accept that if anything is to be done, it is the patient who must do it. The boy has to help himself and learn to cope with waves. The psychotherapist does not play King Canute.

